# Individual and community level factors with a significant role in determining child height-for-age Z score in East Gojjam Zone, Amhara Regional State, Ethiopia: a multilevel analysis

**DOI:** 10.1186/s13690-017-0193-9

**Published:** 2017-05-05

**Authors:** Zewdie Aderaw Alemu, Ahmed Ali Ahmed, Alemayehu Worku Yalew, Belay Simanie Birhanu, Benjamin F. Zaitchik

**Affiliations:** 1 Public Health Department, College of Health Sciences, Debre Markos University, P.O. Box 269, Debre Markos, Ethiopia; 2 School of Public Health, College of Health Sciences, Addis Ababa University, P.O.Box 14 575, Addis Ababa, Ethiopia; 3 Center for Environment and Development, College of Development Studies, Addis Ababa University, P. O. Box 56649, Addis Ababa, Ethiopia; 4 Department of Earth and Planetary Sciences, Johns Hopkins University, Baltimore, USA

**Keywords:** Stunting, Children, Multilevel, East Gojjam Zone, Amhara Regional State, Ethiopia

## Abstract

**Background:**

In Ethiopia, child undernutrition remains to be a major public health challenge and a contributing factor for child mortality and morbidity. To reduce the problem, it is apparent to identify determinants of child undernutrition in specific contexts to deliver appropriately, targeted, effective and sustainable interventions.

**Methods:**

An agroecosystem linked cross-sectional survey was conducted in 3108 children aged 6–59 months. Multistage cluster sampling technique was used to select study participants. Data were collected on socio-demographic characteristics, child anthropometry and on potential immediate, underlying and basic individual and community level determinants of child undernutrition using the UNICEF conceptual framework. Analysis was done using STATA 13 after checking for basic assumptions of linear regression. Important variables were selected and individual and community level determinants of child height-for-age Z score were identified. *P* values less than 0.05 were considered the statistical level of significance.

**Results:**

In the intercept only model and full models, 3.8% (*p* < 0.001) and 1.4% (*p* < 0.001) of the variability were due to cluster level variability. From individual level factors, child age in months, child sex, number of under five children, immunization status, breast feeding initiation time, mother nutritional status, diarrheal morbidity, household level water treatment and household dietary diversity were significant determinants of child height for age Z score. Also from community level determinants, agroecosystem type, liquid waste disposal practice and latrine utilization were significantly associated with child height-for-age Z score.

**Conclusion:**

In this study, a statistical significant heterogeneity of child height-for-age Z score was observed among clusters even after controlling for potential confounders. Both individual and community level factors, including the agroecosystem characteristics had a significant role in determining child height-for-age Z score in the study area. In addition to the existing efforts at the individual levels to improve child nutritional status, agroecosystem and community WASH related interventions should get more attention to improve child nutritional status in the study area.

## Background

Height-for-age Z-score is used as an indicator of linear growth retardation and cumulative growth deficits in children under 5 years. A child with a height-for-age Z score (HAZ) less than minus two standard deviations (<−2 SD) below the median of a reference height-for-age standard deviations is considered as stunted [1].

Globally, the prevalence of child stunting has shown reduction from 47% in 1985 to 23.2% in 2015 [2]. In Africa, childhood stunting reduced from 38% in 2000 to 32% in 2016 [2]. Similarly in Ethiopia, childhood stunting prevalence shows a steady decline from 58% (in 2000) [3] to 38.4% (in 2016) [4]. However, still child undernutrition including childhood stunting remains to be a major public health challenge [5], contributing to child mortality and morbidity, including in Ethiopia [6].

In Ethiopia, fifty-seven percent of all child deaths are related to undernutrition and contribute to 28% of all child mortality [7]. In the country, 16% of all repetitions in primary schools, 8% work force reduction and loss of 55.5 billion Ethiopian Birr per year (16.5% of the GDP) are implicated to be undernutrition [5]. Also, child undernutrition elongates school enrolment age [8], limits growth and development of young children and infants [9–11], lowers cognitive and academic performance, affects psychosocial interaction, elevates experience of anxiety, depression and other symptoms of common mental disorders [12, 13].

To reduce burden of child undernutrition and to deliver appropriate, effective and sustainable solutions to the problem and to meet the needs of the most vulnerable people [14], recognition of determinants in specific contexts is very crucial. The Food and Nutrition Security framework developed by UNICEF recognizes three levels of determinants of undernutrition: the basic, underlying and immediate causes of undernutrition [15]. The most important immediate determinants contributing to children’s undernutrition, include the disease burden and dietary intake [15]. The underlying factors of child undernutrition, include household food insecurity, environmental health conditions and maternal and child care practices [15]. The basic causes, include health service accessibility, population educational status, gender roles, marital status, fertility related factors, child age and gender, economical factors and agroecosystem characteristics [15].

In the Ethiopian context, studies were done to identify determinants of child undernutrition in different parts of the country [4, 16, 17]. Most of those studies did their analysis using single level regression, ignoring the presence of neighborhood effects of predictors (externalities), presence of community level factors and hierarchical nature of the sampling procedures [18]. Trying to estimate the determinants using a single level of analysis gives incomplete picture to understand the true association of child undernutrition and determinants. Therefore, appropriate methodology is required for more comprehensive and accurate analysis of determinants of child undernutrition, using multilevel modeling which is designed to overcome the above difficulties by treating variables based on their levels [18].

In addition, most of the above studies did not assess the effect of agroecosystem on child undernutrition systematically. An agroecosystem which vary by agro climatic factors, like precipitation and temperature, soil and terrain analysis and the farming system have potential to affect child nutritional status [19]. The influence of agroecosystem can be observed through multiple pathways, including affecting dietary intake through its influence on food production in terms of quantity and quality, disease distribution, due to water and sanitation services and maternal and child care services through geographic and financial accessibility of health services [20]. So, analysis of child undernutrition determinant factors, including agroecosystem as community level factor is very important to target nutrition specific and nutrition sensitive interventions.

Therefore, this study was planned to identify individual and community level determinants of child height for age Z score which helps to understand the contribution of individual and community level factors, including agroecosystem characteristics. Such findings as well provide relevant and pertinent information to design interventional strategies and programs, taking in to account the community level variations.

## Methods

### Study area and period

This study was conducted in East Gojjam Zone, Amhara Regional State, Ethiopia. The area consists of different climatic zones from Choke Mountain (Blue Nile highlands) to Blue Nile depressions [19].

According to the 2015 Amhara Regional Bureau of Finance and Economics Development Report, a total of 381,309 under five children were reported in East Gojjam Zone [21]. East Gojjam Zone has a total of four town administrations and 16 rural districts. The area includes the Choke Mountain watersheds found in the Blue Nile Highlands of Ethiopia, which extends from tropical highland of over 4000 m elevation to the hot and dry Blue Nile Gorge, below 1000 m from sea level [19].

Based on different parameters such as farming system, temperature, rainfall, soil type, climate change vulnerability and climate adaptation potential, the area is divided into six agroecosystem with its respective characteristics [19]. The lowlands of Abay valley (Agroecosystem one) is characterized by low land areas with unfavorable agro ecological conditions with extensive land degradation [19]. The midland plains with black soil (agroecosystem two) is characterized with a considerable high agricultural productivity potential [19]. The midland plains with brown soil (agroecosystem three) is suitable for its agricultural productivity, since it has a good potential to use mechanized agriculture and irrigation schemes [19]. The midland sloping lands with red soils (agroecosystem four) is characterized by low natural fertility with high level of soil acidity, slope terrain and higher rate of water runoff with soil erosion making the crop production potential very low [19]. Hilly and mountainous highlands (agroecosystem five) is found in the hilly and mountainous highlands with constrained crop productivity due to erosion and deforestation [19]. The last agroecosystem is in the top of the mountain with relatively low agricultural productivity, due to its low temperature and since it is a conserved area [19]. This study was conducted from January to April 2015.

### Study design

A multistage stratified cross-sectional survey by agroecosystem was conducted to identify individual and community level determinant factors of child height for age Z score (stunting) among children aged 6–59 months.

### Sample size and sampling procedures

Sample size was calculated using double population formula [22, 23] for means using the mean of height for age Z score across different agro ecological zones. The EDHS 2011 data which contains altitude information above sea level were used to categorize the study clusters in to agro ecological zones of highland, midland and low land. The mean height for age Z score was calculated with standard deviation for each agro ecology zone assuming that agro ecology is one of the main determinants of child undernutrition in the study area. The height for age Z score was calculated for important variables to check the adequacy of the calculated sample size and found that sample size determined using agro ecology was found to be the maximum one.

The computation was made with the following inputs in to Open Epi, Version 3 [24]: height for age Z score of −1.21 for low land areas and −1.40 for highland areas, 95% confidence level (Z _α/2_ = 1.96), design effect of 1.5, 80% power of the study and one to one ratio between at higher risk population (from highland areas) and lower risk population (from lowland areas). Each of the group’s population height for age Z score standard deviations were calculated from the data set and found to be 1.70 and 1.6 for low (lowland areas) and high (high land areas) risk groups, respectively. Then, the calculated sample size was found to be 2379 under five children (1185 for each group). However, to increase the power and apply multilevel model, we used the larger sample size of 3225, which was calculated to address another component of the study. A multistage cluster sampling procedure was used to select those study participants from 38 clusters.

From each agroecosystem, sample districts from the East Gojjam Zone, kebeles from each district and clusters from each Kebele were selected using multistage cluster sampling technique. In the initial phase, five districts which represent one agroecosystem each were selected. Lowlands of Abay valley area (agroecosystem one) was represented by sample taken from Dejene District. The midland plain with black soil area (agroecosystem two) was represented by sample taken from Awabel District. The midland plains, with red soil area (agroecosystem three) were represented by sample from Debre Eliyas District and midland plains, with brown soil area (agroecosystem four) was represented by sample taken from Gozamin District. Finally, the hilly and mountainous area (agroecosystem five) was represented by sample taken from Sinan District.

In the second phase, from each agroecosystem, potential rural kebeles were selected using simple random sampling technique. Since, a district may consist of more than one agroecosystem; care was taken to avoid misclassification bias during kebele selection. List of all kebeles that can represent agroecosystems were listed within a district and then using lottery method, 6–9 were selected randomly. In the third step, from each selected kebele, one got (lowest administrative level) was selected using simple random sampling. The total number of clusters, included were 38 from the five agroecosystems and all eligible under-five children were considered for the survey.

### Data collection

Data were collected by trained data collectors on socio demographic characteristics, using interviewer administered questionnaire and child anthropometry using height and weight measuring scales. In addition, data were collected on the potential immediate, underlying and basic determinants of child undernutrition, using interviewer administered questionnaire. From the immediate determinants, data were collected on childhood illness 2 weeks prior to the survey and on dietary intake data, including breast feeding practice, complementary feeding practice, child dietary diversity, and maternal nutritional status. The underlying determinants of child undernutrition, including household food insecurity, environmental health conditions and maternal and child care practices data were collected. Latrine utilization was assessed using four main indicators as a check list: presence of foot path to the latrine, not using the latrine as store, presence of fresh feces around the hole of the latrine and absence of feces around the compound. Also household level waste management practice was assessed using a check list on the presence of the pit and current utilization.

Household food insecurity status was measured using Household Food Insecurity Access Scale (HFIAS) of Food and Nutrition Technical Assistance (FANTA) questionnaire developed in 2006 with a recall period of 30 days. Observational check lists were used to collect data on Environmental health conditions. Also maternal health service utilization, wealth status, and child care practice data were collected using interviewer administered questionnaire. Agroecosystem type data were accessed using previously published article on the area [25].

Weight of the child was measured using a digital scale designed and manufactured under the guidance of UNICEF with 100 g precision to measure body weight. Length/height measurements were taken using a locally produced UNICEF measuring board with a precision of 0.1 cm. Children below 24 months of age were measured in a recumbent position, while standing height was measured for those who were 24 months and older. Weight and height of the child were taken twice and variations between the two measurements of 100 g were accepted as normal for weight and 0.1 cm in height/length of the children. However, repeated measurements were carried out upon significantly larger variations which were above 100 g in weight and 0.1 cm in height/length. The weight scale was calibrated before measuring the child weight (Table 1).

**Table 1:** Definition and measurements of variables used in the dissertation, East Gojjam Zone, Amhara Regional State, Ethiopia, 2015

Variable	Measurement of variable
Dependent variable	
Child Height for Age Z score	Height-for-age Z-score (stunting) is an indicator of linear growth of a child and stunting refers to a child who is too short for his or her age.
Individual level factors	
Child gender	Categorized in to (0) Boy; (1) Girl
Child age in months	Child age was measured in months
Number of under five children	Categorized in to (0) one; (1) two or more
Educational status of the mother	Categorized in to (0) cannot read and write, (1) only can read and write, but without formal education, (2) have formal education.
Women participation in decision making	Categorized in to (0) No; (1) Yes
Household Wealth status	Categorized in to (0) poorest quintile; (1) second quintile; (2) third quintile; (3) fourth quintile; (4) richest quintile.
Child Immunization status	Categorized in to (0) not fully immunized; (1) fully immunized
Childhood diarrheal illness	Categorized in to (0) No; (1) Yes
Time of breast feeding initiation	Categorized in to (0) after 1 h; (1) within an hour
Complementary feeding initiation time	Categorized in to (0) before 6 months; (1) at 6 months; (2) after 6 months.
House hold diversity score	Measured using 12 food groups household consumption of 24 h prior to the survey
Household food insecurity score	Measured using FANTA food access scale and it ranges from zero (food secure) to 27 (severely food insecure).
Mother MUAC^a^	Categorized in to (0) < 21 cm; (1) ≥ 21 cm
Antenatal Care (ANC)	Categorized in to (0) No; (1) Yes
Postnatal Care (PNC)	Categorized in to (0) No; (1) Yes
Household level water treatment	Categorized in to (0) No; (1) Yes
Community level factors	
Latrine utilization	The four indicators were categorized in to (0) No; (1) Yes. Yes refers to a household who satisfied the four indicators.
Household waste management practice	Categorized in to (0) No; (1) Yes. Yes refers to households who have disposal site and used during the time of survey.
Agroecosystem type	Categorized in to (0); Low lands of Abay valley (1) Midland with black soil; (2) midlands with red soil; (3) midlands with brown soil (4) hilly and mountainous highland.

^a^ *MUAC* Mid Upper Arm Circumference

### Quality control

Intensive training with pretesting was given for 5 days to data collectors and supervisors to ensure all research team members can administer the questionnaires properly, read and record measurements accurately. The pretesting was done in none selected with have similar characteristics to the study community. Then, all necessary corrections were made based on the pretest, before the actual data collection. Repeated measurements were taken during weight and height measurements to check the consistency of measurements by two measurers independently. At the end of every data collection day, each questionnaire was examined for its completeness and consistency and pertinent feedbacks were given to the data collectors and supervisors to correct it in the next data collection day. Data were cleaned using frequencies for logical and consistency errors before further analysis.

### Data management and analysis

Data were cleaned, coded and entered into EPI Info version 3.5 [26] and exported to STATA (Stata Corp LP, College Station TX) [27]. Child nutritional status was calculated using WHO Anthro version 3.2.2 [28]. Since the outcome is a continuous variable, normal distribution of the dependent variable assumption was checked using graphical and formal statistical tests. Multicollinearity was checked using the variance inflation factor (VIF) and variables with VIF less than 10 were considered for the analysis. Descriptive statistics was used to present frequencies, with percentages in tables and using texts. Multilevel linear mixed effects regression analysis was used to identify individual and community level factors after selecting important variables using simple linear regression analysis using *P* < 0.05 as an entry criteria to the model.

As justifications for using a multilevel linear modeling is related to the determinants of child stunting are found at different level and factors some have neighborhood effects which impose negative/positive externality in the surrounding community [29, 30]. As a result analyzing variables from different levels at one single common level using the classical linear regression model leads to bias (loss of power or Type I error). This approach also suffers from a problem of analysis at the inappropriate level (atomistic or ecological fallacy). Multilevel models allow us to consider the individual level and the group level in the same analysis, rather than having to choose one or the other. Secondly, due to the multistage cluster sampling procedure in the current study, individual children were nested within clusters/villages/; hence, the probability of a child being stunted is likely to be correlated to the cluster level factors. As a result, the assumption of independence among individuals within the same cluster and the assumption of equal variance across clusters are violated in the case of nested data. Hence, the multilevel analysis is the appropriate method for such kind of studies [29, 30].

Assuming the continuous responses variable Y_ij_ depend on individual level explanatory variable X_ij_ and community level explanatory variable Z_j_, if deviation from the average intercept and slope due to cluster (community level factors) effect are represented by u_0j_ and u_1j_, the two models are given in the following way. The intercept-only model = Y_ij_ = γ_00_ + u_0j_ and the full model = Y_ij_ = γ_00_ + γ_01_Z_j_ + γ_10_X_ij_ + u_0j_ + u_1j_X_ij_. The intercept γ_00_ and slopes γ_01_ and γ_10_ are fixed effects, whereas u_oj_ and u_1j_ are random effects of level-2. The intercept-only model allows us to evaluate the extent of the cluster variation influencing child height for age Z score [31]. The intra-class correlation coefficient (Rho) refers to the ratio of the between-cluster variance to the total variance and it tells us the proportion of the total variance in the outcome variable that is accounted at cluster level.

Mathematically, Rho (ICC), is given as $\uprho =\frac{\delta_o^2}{\delta_o^2+{\delta}^2}$ Where σ^2^ refers to individual level variance and σ_0_^2^ refers to community level variance. The regression coefficients were interpreted and *p* values less than 0.05 were considered as level of significance [31].

## Results

### Socio-demographic characteristics

From the total 3210 study participants, 3108 were considered for analysis which makes the response rate 96.8%. As indicated in Table 2 below, 1565 (50.4%) of the children were females. The mean age of children was 29.2 (±14.7) months and 853 (27.4%), 723 (23.3%) and 710 (22.8%) were in the age range of 12–23, 24–35 and 36–47 months, respectively. In the study, the mean age of the mother during birth of the index child was 30.76 (±6.3) years and 29.3% were in the age range of 25–29 years and 25.5% were in the age range of 30–34 years.

**Table 2:** Socio demographic characteristics of study participants in East Gojjam Zone, Amhara Regional State, Ethiopia, 2015

Variables	Category	Frequency	Percentage
Child sex	Male	1543	49.6
	Female	1565	50.4
Child age	6–11 month	424	13.6
	12–23 month	853	27.4
	24–35 month	723	23.3
	36–47 month	710	22.8
	48–59 month	398	12.8
Household head sex	Male	2851	91.7
	Female	257	8.3
Mother marital status	Married	2823	90.0
	Divorced	187	6.0
	Separated	67	2.2
	Widowed	31	1.0
Religion	Orthodox	3096	99.6
	Other^a^	12	0.4
Ethnicity	Amhara	3102	99.8
	Other^b^	6	0.1
Father Education	No formal education	1490	47.9
	Primary (1–6 grade)	1202	38.7
	Secondary and above	416	13.4
Mother education	No formal education	2492	80.2
	Primary (1–6 grade)	304	9.8
	Secondary and above	312	10.0
Mother occupation	Farmer	2856	91.9
	House wife	99	3.2
	Merchant	77	2.48
	Daily Laborer	36	1.2
	Employed	16	0.5
	Other^c^	24	0.8
Mother age in years	14–19	44	1.4
	20–24	387	12.5
	25–29	906	29.3
	30–34	790	25.5
	35–39	650	21.0
	40–44	225	7.3
	45–49	99	2.9
Average family size	<5 members	1589	51.1
	≥5 members	1519	48.9
Number of under five children	One	2593	83.4
	Two	515	16.6
Women participation in household decision	Yes	2683	86.3
	No	425	13.7

^a^Muslim and protestant, ^b^Oromo and Tigre, ^c^Carpenter and pottery

In the study, majority (91.7%) of household heads’ were males, married (90%), Orthodox Christians (99.6%) and Amhara (99.8%). The study indicated that 2492 (80.2%) of the women and 1490 (47.9%) of the men have no formal education and 2683 (86.3%) women participated in household decision making activities. Majority of mothers (91.9%) and fathers (93.1%) of the children were farmers in their occupation. In the study, 2593 (83.4%) households have only one child and 1589 (51.1%) have a family size of five and above.

### Diet intake, child and maternal care and environmental health conditions

As indicated in the Table 3 below, 2196 (70.7%) of the mothers initiated breast feeding to the infant within an hour of delivery and 1600 (51.5%) of mothers initiated complementary feeding on the recommended time of 6 months. In the study, 648 (20.8%) mothers Mid Upper Arm Circumference (MUAC) was less than 21cms. In the last 2 weeks of the survey, 407 (13.1%) of children had diarrheal illness.

**Table 3:** Diet intake, child and maternal care practices and Environmental health condition related characteristics of study participants in East Gojjam Zone, Amhara, Regional State, Ethiopia, 2015

Variables	Category	Frequency	Percentage
Breast feeding initiation time	Within one hour	2196	70.7
	1–24 h	718	23.1
	After 24 h	104	3.3
	Not breast feed	90	2.9
Complementary feeding initiation time	<6 months	282	9.1
	at 6 months	1600	51.5
	>6 months	1226	39.4
Maternal MUAC	<21 cm	648	20.8
	>21 cm	2460	79.2
Diarrheal illness 2 weeks prior to the survey	Yes	407	13.1
	No	2701	86.9
Household food insecurity	Food secure	1077	34.7
	Food insecure	2031	65.3
Latrine utilization^a^	Yes	714	23.0
	No	2395	77.0
Liquid waste disposal practice	Yes	1132	36.4
	No	372	12.0
ANC follow up^b^	Yes	2412	77.6
	No	696	22.4
PNC follow up^c^	Yes	1138	36.6
	No	1970	63.4
Child immunization status	Incomplete	2522	81.1
	complete	586	18.9

^a^ *MUAC* Mid Upper Arm Circumference, ^b^ *ANC* Antenatal Care, ^c^ *PNC* Postnatal Care

In this study, 2031 (65.3%) of the households were food insecure. In this survey, 714 (23%) of the households’ used latrine and 2736 (88%) of the households practiced liquid waste disposal. In the study, 2412 (77.6%) of the mothers attended at least one ANC follow up and 1138 (36.6%) had PNC follow uo within 5 days of delivery. The full immunization status of the child was 18.1% in the study area.

The overall prevalence of child stunting was 39.0% (37.32, 40.75) and the prevalence of childhood stunting among males was 41.7 and 36.4% for females, respectively. Child stunting increased as the age of the child increased. The maximum child stunting was observed in the age range of 48–59 months (63.8%) and the minimum was from age range of 6–11 months (21.9%).

### Factors associated with childhood height for age Z score

As indicated in the empty model of the multilevel mixed effects linear regression analysis in Table 4, from the total variation across communities (clusters), 3.8% (*p* < 0.001) of the variance is attributable to cluster level variance, which suggests the need for multilevel mixed effects linear regression analysis rather than using the traditional linear regression analysis.

**Table 4:** Multilevel regression analysis results on individual and community level determinants of child stunting in East Gojjam Zone, Amhara Regional State, Ethiopia, 2015

Measure of variation	Model 1^a^	*P* value	Model 2^b^	*P* value	Model 3^c^	*P* value	Model 4^d^	*P* value
Level 2 variance (SE)	0.16	<0.001	0.08	<0.001	0.06	<0.001	0.04	<0.001
Level one variance (SE)	4.04		2.88		3.89		2.85	
ICC (%)	3.8%		2.7%		1.9%		1.4%	
Model fit statistics DIC (−2loglikelihood)	6606		6077		6538		6054	

^a^Model 1 is the intercept only model which is a baseline model without determinant variable. ^b^Model 2 is adjusted for individual level factors. ^c^Model 3 is adjusted for community level factors. ^d^Mode4 is the final model adjusted for both individual and community level factors. *ICC* intracluster correlation, *DIC* deviation information criteria

In model two, after adjustment for level one factors, the variance attributable to cluster level was reduced to 2.7% (*P* < 0.001). Similarly, after controlling level two factors in model three, the variance attributable to cluster level was reduced to 1.9%. Finally, in model four, after adjusting for both individual and community level factors at the same time, only 1.4% (*p* < 0.001) of the variance is attributable to community level variance.

#### Individual level determinant factors

As indicated in Table 5 below, child sex and age were independent predictors of child height-for-age Z score. When, on average, the child’s age increased by 1 month, the mean height-for-age Z score decreased by −0.04 (*P* < 0.001) and being female child improved the child mean height-for-age Z score by 0.16 (*P* < 0.01) compared to males. Number of under-five children in the household showed a statistical significant association with child means height-for-age Z score. A child from households with two or more under five children, the mean height-for-age Z score decreased by −0.48 (*P* < 0.001) compared to children from households with one child.

**Table 5:** Factors Associated with child height for age Z score in Ethiopia by multilevel linear regression analysis, 2015

Variables	Model 2^a^ ß**(SE)	*P* value	Model 3^b^ ß** (SE)	*P* value	Model 4^c^ ß** (SE)	*P* value
Level one factors						
Child age in months	−0.04 (0.002)	*P* < 0.001			−0.04 (0.002)	*P* < 0.001
Child sex (male^d^)						
Female	0.16 (0.06)	0.008			0.16 (0.06)	0.009
Number of under-fives in the household (one^d^)						
Two and above	−0.48 (0.08)	*P* < 0.001			−0.48 (0.08)	*P* < 0.001
Mother education (can’t read and write^d^)						
Only read and write	0.04 (0.10)	0.70			0.004 (0.10)	0.97
Have formal education	0.13 (0.11)	0.21			0.14 (0.11)	0.20
Wealth index (Lowest^d^)						
Second	0.09 (0.11)	0.41			0.08 (0.11)	0.50
Middle	0.09 (0.11)	0.43			0.06 (011)	0.60
Fourth	0.01 (0.12)	0.96			0.02 (0.12)	0.85
Highest	0.03 (0.08)	0.70			0.05 (0.08)	0.53
Women participation in decisions (No^d^)						
Yes	0.11 (0.09)	0.24			0.09 (0.09)	0.35
ANC follow up (No^d^)					0.05 (0.08)	0.51
Yes	0.05 (0.08)	0.56				
PNC follow up (No^d^)					0.06 (0.07)	0.39
Yes	0.09 (0.07)	0.24				
Child immunization (Not immunized^d^)						
Fully immunized	1.35 (0.08)	*P* < 0.001			1.30 (0.09)	*P* < 0.001
Breast feeding initiation time (within an hour^d^)						
After an hour	−0.30 (0.07)	*P* < 0.001			−0.25 (0.07)	0.001
Complementary feeding initiation (on time^d^)						
Not on recommended time	−0.10 (0.06)	0.11			−0.10 (0.06)	0.13
Maternal MUAC in cm	0.12 (0.01)	*P* < 0.001			0.12 (0.01)	*P* < 0.001
Household food insecurity access score	−0.01 (0.01)	0.11			−0.01 (0.01)	0.20
House hold diversity score	0.07 (0.03)	0.01			0.07 (0.03)	0.01
Household level water treatment (No^d^)						
Yes	0.18 (0.10)	0.07			0.25 (0.10)	0.01
Diarrhea illness 2 weeks prior to the survey (No^d^)						
Yes	−0.27 (0.09)	0.003			−0.29 (0.09)	0.001
Community level factors						
household waste disposal practice (No^d^)						
Yes			0.26 (0.11)	0.01	0.20 (0.10)	0.04
agroecosystem type (Abay valley lowlands)						
Midland with black soil			0.60 (0.19)	0.01	0.52 (0.15)	0.001
Midland with red soil			0.48 (0.19)	0.001	0.32 (0.16)	0.04
Midland with brown soil			0.51 (0.18)	0.006	0.50 (0.15)	0.001
Hilly and mountainous highlands			0.09 (0.18)	0.61	0.18 (0.16)	0.27
Latrine utilization (No^d^)						
Yes			0.84 (0.08)	*P* < 0.001	0.39 (0.07)	*P* < 0.001

^a^Model 2 is adjusted for individual level factors. ^b^Model 3 is adjusted for community level factors. ^c^Mode4 is the final model adjusted for both individual and community level factors

^d^Indicates the reference category, ß** it refers to normal regression coefficient

When the child was fully immunized, its height-for-age Z score increased by 1.23 (*P* < 0.001) compared to a child who was not fully immunized. Initiating breast milk feeding after an hour, height-for-age Z score decreased by −0.25 compared to those children where breastfeeding started within an hour of delivery time. Childhood diarrhea illness showed significant association with child height-for-age Z score. When children had diarrheal episode in the last 2 weeks of the survey, their mean height-for-age Z score decreased by −0.29 (*P* < 0.001) compared to children without diarrheal illness.

In this study, maternal undernutrition showed a negative significant association with child height-for-age Z score. When the mother Mid Upper Arm Circumference (MUAC) increased by one centimeter, child height-for-age Z score increased by 0.12 (*P* < 0.001). The study demonstrated that if household dietary diversity score increased by one food group, the child height-for-age Z score increased by 0.07 (*p* < 0.01). Household food insecurity access score did not show a statistical significant association with child Height-for-Age Z score. Household level water treatment practice has improved child height-for-age Z score by 0.25 (*p* < 0.05) compared to households that did not treat water at household level. Child Mother antenatal care follows up and postnatal care services utilization did not show a statistical significant association with child height-for-age Z score.

#### Community level determinant factors

As indicated in Table 5 below, the household waste management practice showed statistical significant association with height-for-age Z score. Households that dispose household waste properly, the mean height for age Z score increased by 0.20 (*p* < 0.05) and households that use the latrine increased child height-for-age Z score by 0.39 (*p* < 0.001) compared to households that did not use the latrine. Agroecosystem characteristics showed a statistical significant association with child height for age Z score. A child from midland with brown soil, midland red soil and midland black soil, whose height for age Z score increased by 0.5 (*P* < 0.01), 0.32 (*P* < 0.05) and 0.52 (*P* < 0.01) respectively, compared to children from low lands of Abay valley. However, a child from hilly and mountainous agroecosystem, whose height for age Z score did not show a statistical significant difference compared to a child from lowlands of Abay Valley.

## Discussion

This study assessed the effect of individual and community level determinants of child height-for-age Z score using multilevel mixed effects linear regression. The finding indicated that child stunting depended on the joint effect of individual and community level determinants. The unexplained variations which are attributable to cluster level factors decreased in the empty model, compared to the full model when we control both level one and level two determinants.

The current study illustrated a negative significant statistical association between child age and height-for-age Z score. Possibly, it might be related as age increases, the child starts to move independently and this may expose children to infections, decrease mother to child frequency of contact to give care, including breast milk and breast milk cannot have nutritional variety, proportion, and suitable balance for the child as age increases [32].

This study demonstrated lower mean height-for-age Z score among males, compared to females in contradiction with what many authors have expected since lower priority of girls in many cultures would bias food consumption towards boys [33]. The possible explanation might be related to higher proportion of preterm and low weight births being common in males compared to females [34, 35]. Another possible explanation for this might be related with childhood morbidity being higher among males than females in early life, even after adjusting for potential confounders [36]. However, there are also studies which showed that height-for-age Z score was not significantly associated with child sex [37, 38] and this mixed result needs more clarification, using advanced epidemiological study designs in local contexts to target specific interventions.

Maternal education has a potential in determining child height-for-age Z score through generating higher incomes, increase information access about child health and nutrition [33], enhancing the mothers’ general knowledge to allocate more resource regarding to children’s wellbeing and care for the child [39]. Studies from India, three Asian republics, Mozambique [39–41], Kenya [42] and Ethiopia [43] indicate that maternal education has a positive influence to improve height-for-age Z score.

Evidences from India [40], Nigeria [44], Kenya [42], Cambodia [45] and Ethiopia [46, 47] indicate that economic status of the household has the potential to improve child height-for-age Z score through accessing nutritious foods at the household level (56). However, in the current study, household wealth index did not show statistical significant association with child height-for-age Z score. The possible explanation for this disparity might be related with poor intra household food distribution among household members [48], where mothers and children receiving a smaller share of the family’s food relative to their nutritional need and simply feeding on a common kind of food, prepared from cereals and pulses [49]. In addition, consumption of balanced diet, including animal products in the study area is very low, except on holydays and festivals [49].

Literature indicates that mothers of children who have ANC visit during pregnancy can get information on child and maternal feeding practices, child care practices and health service use [18]. Similarly, studies from Ethiopia indicate that ANC follow up has a positive contribution to improve child height-for-age Z score [43, 50]. However, this study demonstrated that ANC follow up of the mother during pregnancy did not show statistically significant association with child height-for-age Z score. In addition, postnatal follow up of the mother has contributions to improve child feeding and care practices [18, 50, 51]. This variation might be related with mother ANC and PNC follow up alone might not be adequate to improve child nutritional status and its effectiveness depends on quality of information given to the mother about child care and feeding practices during the follow up.

In the current study, mother nutritional status showed a statistical significant association with child height-for-age Z score which is supported by studies from Kenya [42], Cambodia [45] and Ethiopia [46]. Initiating breast milk feeding within an hour of delivery [52] contributes to improve child height-for-age Z score. It is believed that appropriate complementary feeding promotes growth and prevents lower child height-for-age Z score [52]. However, in the current study time of complemnentary feeding did not show a statistical significant association with child height-for-age Z score wich is not in line with a study from West Gojjam Zone [53]. This might be related to the fact that, with the existence of different factors contributing to child undernutrition, time of intiation of complementary feeding alone might not reduce the problem to the expected level which suggests the importance multiple interventions to bring about a remarkable change.

Child immunization status has a strong positive link with child better nutritional status [54] and this study indicated that child full immunization status showed a positive statistical significant association with child height-for-age Z score. A similar finding was reported from studies done from Ethiopia [50, 55] Kenya [56], Somalia [57] and Nigeria [58]. This study demonstrated that childhood diarrheal illness in the last 2 weeks of the survey has a negative association with child height-for-age Z score which is supported by studies from Ethiopia [16, 50] and Cambodia [45].

The other important finding of this study was that better household dietary diversity score improved child height-for-age Z score significantly, which is supported by a study from the Amhara Regional State [49]. This is related with the fact that dietary diversity ensures nutrient adequacy [59] and it is a proxy indicator of micronutrient adequacy of human diet [60]. Household food insecurity is one of the key determinants of nutritional status of children [44, 50, 51, 61]. However, in the current study, household food insecurity access score did not show statistical significant association with child height for age Z score, which is supported by studies from Colombia [62], Pakistan [63] and Ethiopia [64]. The possible explanation of the result might be related with contribution of food security on child nutritional status affected by intra household food distribution culture [48] and consumption of balanced diet, including animal products, the availability is very low in the country and the study area the exception of holydays and festivals.

Community water sources safety is recognized as an essential component to improve child undernutrition [33] through reducing frequent infection [16]. In addition, household level water treatment and safe handling during transportation, storage or consumption is very important i.e. water, sanitation and Hygiene (WASH) measures. This study indicates that household level water treatment improves child height for age Z score.

This study demonstrated that latrine utilization showed a positive statistically significant association with child height-for-age Z score. The current finding contradicts with a study in Ethiopia that indicated latrine availability for the household did not show a significant association with child height-for-age Z score [16]. The possible explanation for the difference might be related to measurment; in the previous study, only physical availability of latrine was assessed, whereas in the current study, latrine utilization was measured using the four WHO recommended indicators [65]. The proper management of household wastes generated from individual houses is very important parts of environmental health services in the community. Improper handling and management of wastes may create breeding places for different vectors and children may develop infection. In the current study, disposing the household liquid waste anywhere showed negative statistical significant association with child height-for-age Z score.

In the study area, lower child nutritional status was expected in the low lands of Abay Valley and hilly and mountainous highland agroecosystem, due to low productivity in relation to higher climate change vulnerability and lower climate change adaptive capacity compared to midland plains with brown, midland sloping land with red soil and midland plains with black soil [19, 25]. This condition has the potential to influence child nutritional status through different pathways, like affecting food security status, disease burden, dietary diversity and other socio economic conditions. The current study also supported the above hypothesis that children from midland plains with brown, midland sloping land with red soil and midland plains with black soil showed better height for age Z score compared to children from low lands of Abay Valley.

The cross-sectional nature of the study has made it difficult to establish cause and effect relationship and could be considered as a limitation of this study. On the other hand, the strength of the study is the use of multilevel linear regression analysis, which overcomes the limitations of the standard regression analysis done before and use of large sample size, with a high response rate which give high statistical power to infer the characteristics of the study population. Environmental health condition data were collected using observational checklist which gives more valid results than simply asking mothers. The study was agro ecosystem linked child nutrition status study which gives more valid result to assess the effect of agroecosystem on child nutritional status.

## Conclusion

In this study, the multilevel linear mixed effects regression analysis identified individual and community level determinants and a significant heterogeneity of child height for age was observed among clusters. The Intra class correlation (unexplained variation) decreased between the empty model (ICC = 3.8%, *P* < 0.001) and full model (ICC = 1.4%, *p* < 0.001) after controlling for both individual and community level determinant factors.

Both individual and community level factors have a significant role in determining child height for age Z score. Policy makers and program managers should understand factors of child height for age Z score that operates both at individual and community levels. Therefore, level-by-level analysis in relation to the determinants of child stunting has a strategic relevance to design appropriate interventions. It can provide very good idea to design and implement child survival service strategies at different levels. Researchers are also advised to conduct further follow up study to evaluate the externalities of the shared environment on child nutritional status.

## Abbreviations

ANC: Ante Natal Care
DIC: Deviance Information Criteria
EDHS: Ethiopian Demographic and Health Survey
FANTA: Food and Nutrition Technical Assistance
HAZ: Height for age Z score
HFIAS: Household Food Insecurity Access Scale
ICC: Intraclass correlation
MUAC: Mid Upper Arm Circumference
PNC: Post Natal Care
SD: Standard deviation
SE: Standard error
UNICEF: United Nations International Children Emergency Fund
VIF: Variance inflation factor
WASH: Water, sanitation and Hygiene
WHO: World Health Organization
